# Gerotranscendence as a Factor of Active Aging: Predictors and Outcomes

**DOI:** 10.1093/geroni/igaa057.1523

**Published:** 2020-12-16

**Authors:** Olga Strizhitskaya

**Affiliations:** Saint-Petersburg State University, Saint-Petersburg, Russia

## Abstract

The image of modern aging had changed. While before ageing was associated with degenerative processes, today more older adults become active and meaningful members of the society. Still more knowledge is needed to help the majority of older adults to age in active and positive way. Solid body of research shows that in ageing the value of subjective factors dramatically increases. Gerotranscendence that suggests important positive personality changes to occur in aging, could be one of such mechanisms. The aim of the present study was to investigate which psychological characteristics were important for development of gerotranscendence and which positive outcomes it might cause. Participants were 200 older adults aged 60-89 (69% females). Methods: Gerotranscendence scale (Strizhitskaya), self-actualization test (Shostrom), Self-acceptance test (Pantileev), Psychological well-being scale (Ryff), Scale of social activity (Strizhitskaya). Our results confirmed in consistence with literature that higher scores on self-acceptance and self-actualization favored development of gerotranscendence. We also found that developed gerotranscendence positively affected psychological well-being. New in our research was that we showed that people who demonstrated higher scores on gerotranscendence were more interested in maintaining social activities; they were interested in participation in social life of their community and were trying to continue active and meaningful social participation. The final model fitted the original data (Chi-square=12,168; df=11; p=0,103; CFI=0,944; GFI=0,973; RMSEA=0,051; PCLOSE=0,503). Thus gerotranscendence had positive effects on older adult’s functioning and social participation.

